# Whole genome sequencing and phylogenetic analysis of African swine fever virus detected in a backyard pig in Mongolia, 2019

**DOI:** 10.3389/fvets.2023.1094052

**Published:** 2023-02-20

**Authors:** Ji-Yeon Hyeon, Erdene-Ochir Tseren-Ochir, Dong-Hun Lee, Sang-Soep Nahm, Douglas P. Gladue, Manuel V. Borca, Chang-Seon Song, Guillermo R. Risatti

**Affiliations:** ^1^Department of Pathobiology and Veterinary Science, University of Connecticut, Storrs, CT, United States; ^2^College of Veterinary Medicine, Konkuk University, Seoul, Republic of Korea; ^3^Department of Infectious Diseases and Microbiology, School of Veterinary Medicine, Mongolian University of Life Sciences, Ulaanbaatar, Mongolia; ^4^Plum Island Animal Disease Center, Agriculture Research Service, US Department of Agriculture, Greenport, NY, United States; ^5^KCAV Co., Ltd., Seoul, Republic of Korea

**Keywords:** African swine fever (ASF), whole genome sequencing (WGS), Mongolia, backyard pig, phylogenetic analysis

## Abstract

African swine fever (ASF) is a highly contagious and fatal disease affecting domestic and wild pigs caused by the African swine fever virus (ASFV). Since the first outbreak in China in August 2018, ASF has spread rapidly in Asia. and the first case in Mongolia was confirmed in January 2019. In this study, we report the first whole genome sequence of an ASFV (ASFV SS-3/Mongolia/2019) detected from a backyard pig in Mongolia in February 2019 using whole genome sequencing. We analyzed their phylogenetic relationship with other genotype II ASFVs from Eurasia. The ASFV SS-3/Mongolia/2019 belonged to genotype II (p72 and p54), serogroup 8 (CD2v), Tet-10a variant (pB602L), and IGRIII variant (intergenic region between the I73R/I329L genes). A total of five amino acid substitutions were observed in MGF 360-10L, MGF 505-4R, MGF 505-9R, NP419L, and I267L genes compared to the ASFV Georgia 2007/1 virus. ML phylogenetic analysis of the whole genome sequence showed that the virus shares a high nucleotide sequence identity with ASFVs recently identified in Eastern Europe and Asia and clustered with the ASFV/Zabaykali/WB5314/2020|Russia|2020 virus which was identified at the border between the Russian Federation and Mongolia in 2020. Our results suggest that trans boundary spread of ASF occurred through close geographic proximity.

## Introduction

African swine fever (ASF) is caused by the African swine fever virus (ASFV), a virus within the genus *Asfivirus* of the family *Asfarviridae*. It is a highly contagious and high-mortality disease affecting domestic pigs and wild boars and a notifiable disease to the World Organization for Animal Health (WOAH) (1, 2). ASF was first described in Kenya in 1921; it subsequently re-emerged from Africa into Georgia and rapidly spread into Eastern Europe and Asia (1). In December 2007, ASF was reported in Russia, with subsequent outbreaks occurring between 2008 and 2009 and affecting domestic pigs and wild boars (1). In August 2018, the disease was reported in China; it spread rapidly across that country and Mongolia, and then to Vietnam, Cambodia, North Korea, Laos, the Philippines, Myanmar, South Korea, Timor-Leste, and Indonesia (2).

On 10 January 2019, Mongolia's State Central Veterinary Laboratory (SCVL) confirmed the first ASF outbreak in Bulgan province, Mongolia (3). After this first detection, 11 additional ASF outbreaks were recorded in Mongolia, involving seven provinces (4). A total of 105 farms and holdings were affected by the disease, resulting in the death or elimination of more than 3,000 exposed pigs (> 10 % of the total pig population in Mongolia) (4). While complete genome sequences of ASFV have been reported from several countries affected by ASF (5–12), a complete genome sequence of ASFV from Mongolia has not been published. However, several characterizations of the ASFV strain from the 2019 outbreak in Bulgan province, Mongolia have been reported in a previous study: partial p72, full p54, partial pB602L, and partial CD2v genes, as well as a 356-bp fragment between the I73R and I329L genes (2).

In this study, we report the first whole genome sequence of an ASFV detected in a backyard pig in Mongolia in February 2019 using next-generation sequencing (NGS). We also analyze its phylogenetic relationship with other genotype II ASFVs from Eurasia.

## 2. Materials and methods

Spleen tissue from a backyard pig carcass found at a dumping ground near Ulaanbaatar was collected during passive surveillance on 14 February 2019, and it was confirmed to be ASFV-positive using quantitative reverse transcription real-time PCR (RT-qPCR) assay (13, 14). DNA was extracted from spleen tissue from the ASF-positive backyard pig carcass using a DNeasy Blood and Tissue kit (Qiagen, Valencia, CA) in accordance with the manufacturer's instructions and eluted in distilled water. DNA concentrations were determined using a Qubit BR dsDNA assay kit (Invitrogen, Carlsbad, CA). DNA samples were diluted to 0.2 ng/μl and libraries were prepared using the Illumina Nextera XT DNA Library Prep Kit (Illumina, San Diego, CA). The concentration of sample libraries was determined using the Qubit dsDNA HS assay kit, and libraries were diluted to a 2 nM concentration and combined in equal volumes to form the pooled library. Subsequently, 600 μl of the 10 pM libraries were submitted for pair-end sequencing using the MiSeq reagent kit v2 (500 cycles) (Illumina, San Diego, CA).

The raw reads were adapter-trimmed for known Illumina adapters and quality-trimmed (*Q* >20 and minimum length >50) with Bbduk (https://sourceforge.net/projects/bbmap). Trimmed reads were mapped to the reference sequence, the ASFV Georgia 2007/1 (GenBank acc. FR682468.2), using minimap2 (15) with the default settings in Geneious Prime 10 Software (https://www.geneious.com/), and the consensus genome sequences (hereafter referred to as ASFV SS-3/Mongolia/2019) were called using Geneious Prime 10 with default parameter settings. To demonstrate the phylogenetic organization of the ASFV, ASFV genotype I 56/Ca/1978 (GenBank acc. MN270969) and all available full-length genome sequences (>150,000 bp) of genotype II ASFVs (*n* = 70) were downloaded from the NCBI GenBank database. The online multiple alignment server MAFFT, version 7 (https://mafft.cbrc.jp/alignment/software/), was used for sequence alignment of whole genome sequences with the default settings (16). Maximum likelihood (ML) phylogenies were constructed using RAxML-HPC v.8 using the general time-reversible (GTR) nucleotide substitution model with gamma distribution and with 1,000 rapid bootstrap replicates (17). Phylogenetic trees were rooted to the ASFV genotype I 56/Ca/1978 as an outgroup and converted to cladogram form for better visualization of the genetic relationships.

## 3. Results and discussion

From 14,542,882 total raw reads, 111,352 reads were mapped to the reference ASF sequence. The length of the assembled genome was 190,591 bp, with a mean coverage depth of 162.3 reads (minimum: 43; maximum: 2,638). The molecular characteristics of ASFV SS-3/Mongolia/2019 were consistent with previous findings on ASFVs from the 2019 outbreaks in Mongolia (2), specifically genotype II (*p72* and *p54*), serogroup 8 (*CD2v*), Tet-10a (*pB602L*), IGRIII variant (intergenic region between the *I73R/I329L* genes). It was found to have a high degree of nucleotide identity (99.98%) with ASFV Georgia 2007/1(data not shown). A total of five amino acid substitutions were observed in genes MGF 360-10L, MGF 505-4R, MGF 505-9R, NP419L, and I267L relative to ASFV Georgia 2007/1 (Supplementary Table 1). MGF360 and MGF505 gene products have been reported to be related to suppression of a type I interferon response, as analyzed using a swine cDNA microarray (18). NP419L encodes DNA ligase involved in DNA replication, repair, nucleotide metabolism, transcription, and other enzymatic activities or host defense evasion (19). Finally, I267L is an important virulence factor that operates by impairing innate immune responses mediated by the RNA Pol-III-RIG-I axis (20).

**Figure 1:**
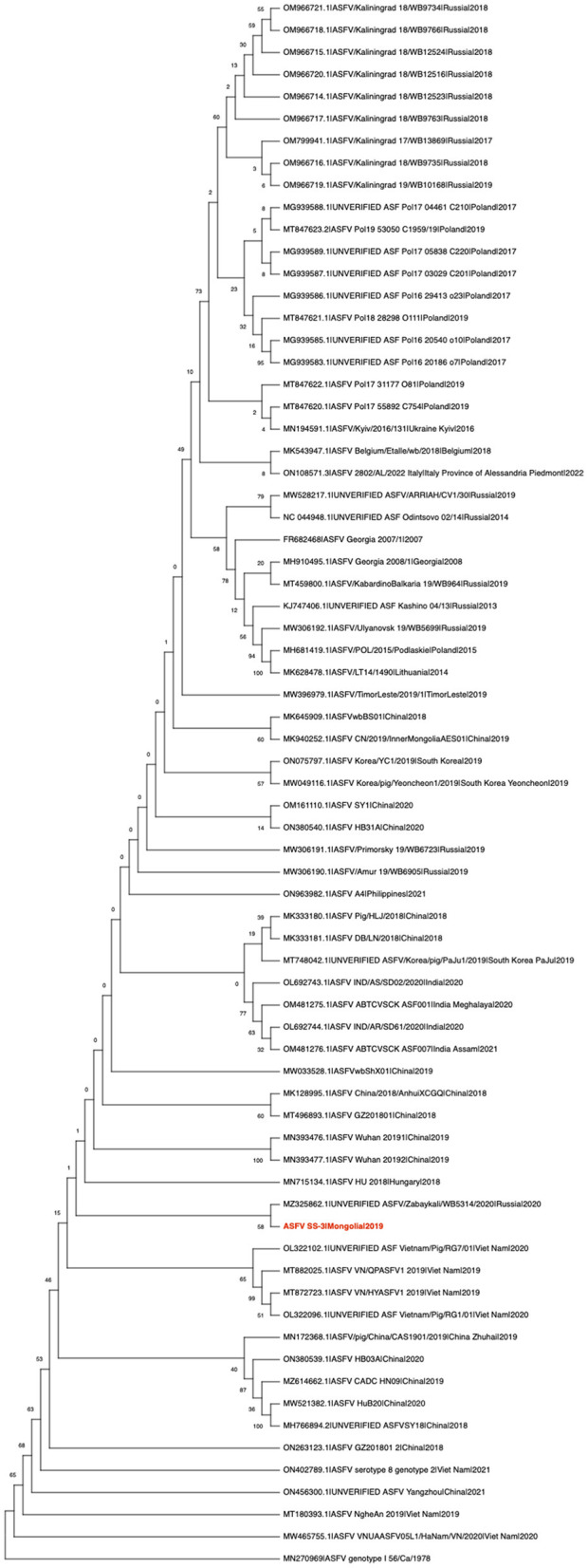
Maximum likelihood analysis of 70 complete coding sequences of ASFV, including the ASFV SS-3|Mongolia|2019 sequenced in this study (in red). The phylogeny was rooted to the ASFV genotype I 56/Ca/1978 as an outgroup and converted to cladogram form for better visualization of the genetic relationships. Numerical values represent 1,000 bootstrap replicate values expressed as percentages.

ML phylogenetic analysis of the whole genome sequence suggested that the virus belongs to the Georgia-07-like genotype II ASF virus (Figure 1). It has a high degree of nucleotide identity (>97%) with ASFVs recently detected in Eastern Europe and Asia (data not shown). It was found to cluster with the ASFV/Zabaykali/WB5314/2020|Russia|2020 virus, which was identified at the border between Russia and Mongolia in 2020 (Figure 1) (5), suggesting that transboundary spread of ASF occurred through close geographic proximity. In late 2020, ASF-related mass mortality of wild boar was observed in MinJiin Khangai mountain, Mungunmorit soum of Tuv province, and Yeroo soum of Selenge province, which are remote areas of Mongolia bordering with Russia. These areas are far from domestic animal farms. We assume that the virus might have been disseminated by the movement of wild boar carriers between Russia and Mongolia.

## Data availability statement

The data presented in the study are deposited in GenBank under the accession number OP787478 and Bioproject accession number PRJNA924538.

## Ethics statement

Ethical review and approval was not required for the study on animals in accordance with the local legislation and institutional requirements.

## Author contributions

J-YH: data analysis and manuscript writing. D-HL: study design, data analysis, and manuscript editing. E-OT-O: data collection and manuscript editing. DG, MB, and GR: study design and manuscript editing. S-SN and C-SS: data analysis, study design, and manuscript editing. All authors contributed to the article and approved the submitted version.
