# Supplementary material for: Whole genome sequencing and phylogenetic analysis of African swine fever virus detected in a backyard pig in Mongolia, 2019
Source: Front Vet Sci. 2023 Feb 20;10:1094052. doi: 10.3389/fvets.2023.1094052 (PMC9986476; doi:10.3389/fvets.2023.1094052)
Supplement: Supplementary file 1 [file Table_1.DOCX]

Supplementary Table 1. Amino acid substitutions found in the ASFV SS-3/Mongolia/2019 virus compared to the ASFV Georgia 2007/1 virus.

| Genes | Position | Amino Acid Change |
| --- | --- | --- |
| MGF 360-10L | 986 | N -> S |
| MGF 505-4R | 757 | H -> Y |
| MGF 505-9R | 967 | K -> E |
| NP419L | 1241 | N -> S |
| I267L | 583 | I -> F |
